# Arabinogalactan Proteins in Plant Roots – An Update on Possible Functions

**DOI:** 10.3389/fpls.2021.674010

**Published:** 2021-05-17

**Authors:** Dagmar Hromadová, Aleš Soukup, Edita Tylová

**Affiliations:** Department of Experimental Plant Biology, Faculty of Science, Charles University, Prague, Czechia

**Keywords:** AGP, arabinogalactan proteins, root growth, root hairs, interactions, fasciclin-like, GPI anchor

## Abstract

Responsiveness to environmental conditions and developmental plasticity of root systems are crucial determinants of plant fitness. These processes are interconnected at a cellular level with cell wall properties and cell surface signaling, which involve arabinogalactan proteins (AGPs) as essential components. AGPs are cell-wall localized glycoproteins, often GPI-anchored, which participate in root functions at many levels. They are involved in cell expansion and differentiation, regulation of root growth, interactions with other organisms, and environmental response. Due to the complexity of cell wall functional and regulatory networks, and despite the large amount of experimental data, the exact molecular mechanisms of AGP-action are still largely unknown. This dynamically evolving field of root biology is summarized in the present review.

## Introduction

Plants are sessile organisms with cells surrounded by cell walls which mediate interactions with surrounding environment. Communication across the cell wall and related cell surface signaling is an essential, complex, and largely unexplored aspect of plant biology (Seifert and Blaukopf, 2010; Duman et al., 2020; Rui and Dinneny, 2020). The deposition and remodeling of the cell wall enables growth and development of plant organs, and cell-wall derived signals mediate responses to internal and external factors (Voxeur and Hofte, 2016; Gigli-Bisceglia et al., 2020).

Arabinogalactan proteins (AGPs) are ubiquitous in the cell wall and in extracellular exudates (Showalter, 2001). They take part in the regulatory and functional continuum of the plasmalemma, cell wall, and environment (Ellis et al., 2010). AGPs occur in all plant organs (Clarke et al., 1979; Fincher et al., 1983; Nguema-Ona et al., 2012; He et al., 2019) but molecular mechanisms of their function remain rather puzzling. They are involved in the regulation of plant growth and development, affect cell wall properties, structure, and architecture (Seifert, 2018, 2021; Tucker et al., 2018), play a role in stem development and differentiation (Ito et al., 2005; MacMillan et al., 2010; Liu et al., 2020), root growth and differentiation (Dolan et al., 1995; Bossy et al., 2009; Nguema-Ona et al., 2012), sexual reproduction (Cheung et al., 1995; Cheung and Wu, 1999; Nguema-Ona et al., 2012; Pereira et al., 2015; Su and Higashiyama, 2018), embryogenesis (Kreuger and van Holst, 1993; Yu and Zhao, 2012; Perez-Perez et al., 2018), fruit ripening (Leszczuk et al., 2020a, b), response to abiotic and biotic stress factors (Mareri et al., 2018; Seifert, 2021), and interactions with microorganisms (Nguema-Ona et al., 2013; Rashid, 2016).

**FIGURE 1:**
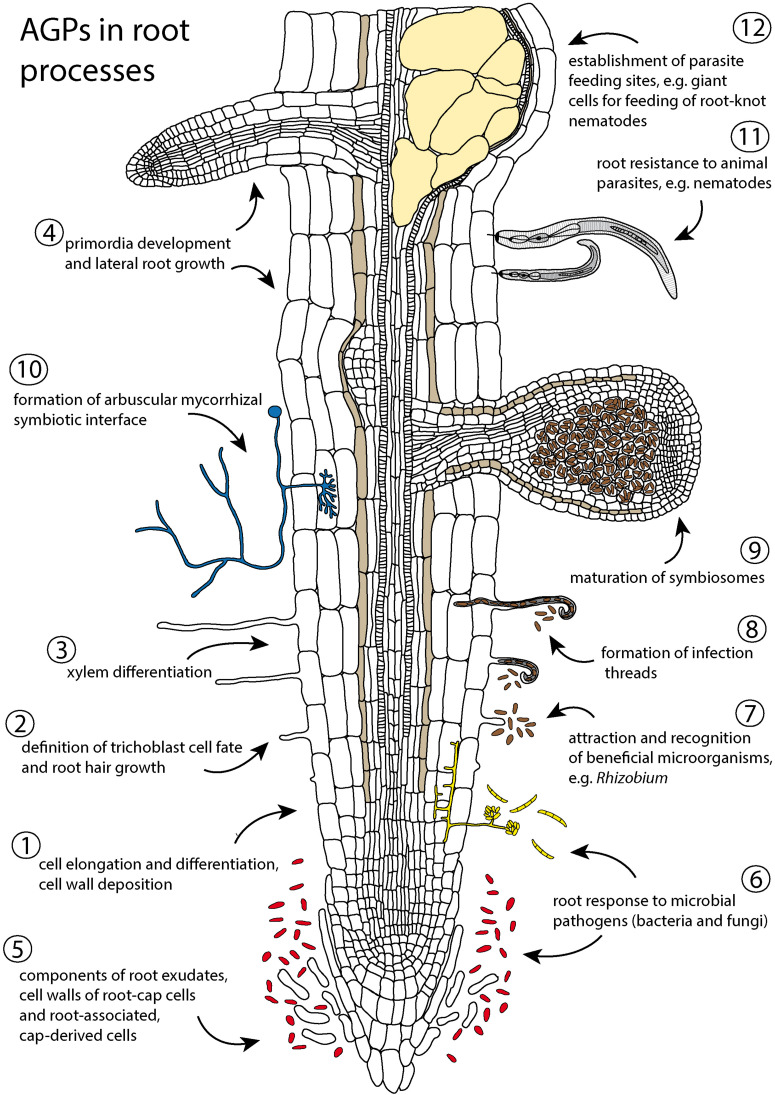
Schematic summary of the involvement of arabinogalactan proteins (AGPs) in root processes. (1–4) AGPs modulate cell wall properties and regulate developmental events in roots: (1) cell division, cell expansion and cell wall deposition (Shi et al., 2003; Yang et al., 2007; Zhang et al., 2011; Seifert, 2018, 2021; Tucker et al., 2018), (2) trichoblast definition and root hair growth (Šamaj et al., 1999; Lin et al., 2011; Marzec et al., 2015; Kirchner et al., 2018; Borassi et al., 2020), (3) xylem differentiation (Dolan et al., 1995; Bossy et al., 2009), and (4) early events of lateral root development (Yang et al., 2007; Johnson et al., 2011; Zhang et al., 2011). (5–12) AGPs are components of root exudates and cell walls of root cap cells and root-associated, cap-derived cells (border cells and border-like cells) and participate in responses to biotic and abiotic environmental factors: (5–6) help to protect roots against abiotic stress (e.g., drought, toxicity) and microbial pathogens (Cannesan et al., 2012; Koroney et al., 2016; Marquez et al., 2018; Driouich et al., 2019; Galloway et al., 2020), (7–10) participate in establishment of mutualistic interaction with N-fixing microorganisms (Berry et al., 2002; Brewin, 2004; Brewin et al., 2008; Tsyganova et al., 2009, 2019; Nguema-Ona et al., 2013), arbuscular fungi (Gollotte et al., 1995; Balestrini and Lanfranco, 2006; Schultz and Harrison, 2008) and beneficial endophytes (Basińska-Barczak et al., 2020; Nivedita et al., 2020), and (11–12) affect root susceptibility to parasites (Beneventi et al., 2013; Bozbuga et al., 2018).

The root system, not covered by a protective cuticle, is constantly interacting with the rhizosphere. It secretes protective mucilage and other compounds, interchanges signaling molecules with soil organisms, and adjusts root development according to the heterogeneous distribution of soil resources with an amazing degree of plasticity. Roots thus present a unique system to evaluate different aspects of AGP functions in the cell wall and extracellular spaces (Figure 1). In roots, AGPs are important regulators of elongation and differentiation of cells (Shi et al., 2003), including root hairs (Kirchner et al., 2018; Borassi et al., 2020). They represent important components of root exudates, aid in the formation of a rhizosheath (Galloway et al., 2020), modulate response to root pathogens and parasites (Gaspar et al., 2004; Bozbuga et al., 2018), and are involved in the establishment of root symbioses with beneficial microorganisms (Brewin, 2004). AGPs even form major components of the glue-like adhesive nanoparticles secreted by the roots of climbing plants (Huang et al., 2016). In this review we summarize selected aspects of AGP action related to root development and function (Figure 1), updating previous excellent reviews (Nguema-Ona et al., 2012, 2013) and covering recent advances in this field of root biology. Available AGP mutants with phenotypic manifestations in roots are summarized (Table 1).

**TABLE 1:** Mutants with modulated expression of *AGP* genes showing phenotypic manifestations in root system.

Gene, locus identifier	Species	Mutant	Gene modulation	Phenotypic manifestations in root system	Important effects in other plant organs
**AGP mutants with observed root phenotypes**					
*AtFLA1*, At5g55730	*A. thaliana*	*fla1*	Knock-out T-DNA	*fla1*:higher number of lateral roots, longer primary roots, altered pericycle cell division on callus inducing medium (Johnson et al., 2011)	*fla1*:reduced shoot regeneration from root explants *in vitro*, no shoot phenotype under normal growth conditions (Johnson et al., 2011)
*AtFLA3*, At2g24450	*A. thaliana*	*fla3*	Knock-down (RNAi)	*fla3*:no root phenotype observed (Li et al., 2010)	*fla3*:shorter siliques, less seeds, abnormal non-viable pollen grains (Li et al., 2010)
		*FLA3*-ox	Overexpression	*FLA3*-ox: enhanced primary root growth, formation of abnormal root cap cells (Li et al., 2010)	*FLA3*-ox: larger leaves, reduced female fertility, very short siliques, less seeds (Li et al., 2010)
*SOS5/AtFLA4*, At3g46550	*A. thaliana*	*sos5/atfla4*	EMS mutag.	*sos5/atfla4*: defective cell expansion, reduced root growth under salinity, altered cell wall structure (Shi et al., 2003), recovered by external ABA (Seifert et al., 2014; Xue and Seifert, 2015)	*sos5/atfla4*: slightly larger leaves, longer petioles, shorter siliques (Shi et al., 2003)
*AtAGP8*, At2g45470	*A. thaliana*	*agp8*	Knock-out (T-DNA)	*agp8*: increased susceptibility to root-knot nematodes (Bozbuga et al., 2018)	*agp8*: not analyzed
*AtAGP14*, At5g56540	*A. thaliana*	*agp14*	Knock-out (T-DNA)	*agp14*: longer root hairs in control and low-Pi conditions (Lin et al., 2011)	*agp14*:not analyzed
*AtAGP15*, At5g11740	*A. thaliana*	*agp15*	Knock-out (T-DNA)	*agp15*: contiguous root hair formation milder then *atagp21* (Borassi et al., 2020)	*agp15*: not analyzed
*AtAGP17*, At2g23130	*A. thaliana*	*rat1/agp17*	Knock-down (T-DNA)	*rat1/agp17*: roots resistant to *Agrobacterium* transformation (Nam et al., 1999; Gaspar et al., 2004)	*rat1/agp17*: not analyzed
*AtAGP18*, At4g37450,	*A. thaliana*	*agp18*	Knock-down (RNAi)	*agp18*: no root phenotype observed (Acosta-García and Vielle-Calzada, 2004)	*agp18*: higher seed abortion (Acosta-García and Vielle-Calzada, 2004)
		*AGP18*-ox	Overexpression	*AGP18*-ox: shorter primary roots, lower number of lateral roots (Zhang et al., 2011)	*AGP18*-ox: abnormal survival of megaspores (Demesa-Arevalo and Vielle-Calzada, 2013), smaller rosettes with multiple branches, less viable seeds, short siliques (Zhang et al., 2011)
*AtAGP19*, At1g68725	*A. thaliana*	*agp19*	Knock-out (T-DNA)	*agp19*: reduced lateral root number, smaller vascular cylinder of primary root (Yang et al., 2007; Zhang et al., 2011)	*agp19*: reduced cell division and expansion in shoot, shorter siliques, less seeds (Yang et al., 2007)
*AtAGP21*, At1g55330	*A. thaliana*	*agp21*	Knock-out (T-DNA)	*agp21*: contiguous root hair formation (Borassi et al., 2020)	*agp21*: not analyzed
*AtAGP30*, At2g33790	*A. thaliana*	*agp30*	Transposon insertion	*agp30*: inhibited initiation of adventitious roots from a callus culture, faster germination, lower sensitivity to external ABA (Van Hengel and Roberts, 2003)	*agp30*: not analyzed
		*AGP30-ox*	Overexpression	*AGP30-ox*: not analyzed	*AGP30-ox*: inhibited shoot development (Van Hengel and Roberts, 2003)
*BcFLA1*	*Brassica carinata*	*bcfla1*	Knock-down (CRISPR)	*bcfla1*: reduced root-hair length in Pi-deficient conditions (Kirchner et al., 2018)	*bcfla1*: not analyzed

*EMS-mutag, selected from ethane-methyl-sulfonate mutagenized population; HSR, high sugar response; MUR, murus; RAT, resistant to agrobacterium transformation; SOS, Salt Overly Sensitive; T-DNA, T-DNA insertion.*

## Arabinogalactan Proteins

Structural proteins are a minor but essential component of the primary cell wall (Rui and Dinneny, 2020) and include proline-rich proteins (PRPs), glycine-rich proteins (GRPs), extensins (EXTs), and AGPs. AGPs are present in vascular plants, bryophytes (Bartels et al., 2017; Johnson et al., 2017; Ma et al., 2017; Classen et al., 2019) and green algae (Palacio-Lopez et al., 2019; Přerovská et al., 2021). AGPs or AGP-like proteins have also been detected in brown algae (Herve et al., 2016) and cyanobacteria (Jackson et al., 2012) opening discussion on their evolutionary origin (Knox, 2016).

Arabinogalactan proteins have the most extensive glycosylation of Pro/Hyp-rich glycoproteins. Their carbohydrate moiety forms 90 to 99% of their molecular mass, combining galactose and arabinose as major sugars with fucose, rhamnose, and glucuronic acid as minor sugars (Fincher et al., 1983; Ellis et al., 2010; Showalter and Basu, 2016; Silva J. et al., 2020). AGPs form a complex family (Showalter, 2001). Their classification has been modified several times over the last decades. Most recently they have been divided into several groups according to their molecular structure: classical AGP, AG peptides, Lys-rich AGPs, chimeric AGPs including FLAs (FASCICLIN-LIKE AGPs), ENODLs (EARLY NODULIN-LIKE AGPs), XYLPs (XYLOGEN-LIKE AGPs), other chimeric AGPs, and HAEs (AGP-EXT hybrids) (Showalter, 2001; Pereira et al., 2015; Mareri et al., 2018; He et al., 2019; Silva J. et al., 2020). Classical AGPs are characterized by the presence of an N-terminal signal sequence, which targets the protein to the endoplasmic reticulum (ER) and secretory pathway, a middle PAST-rich domain (rich in Pro, Ala, Ser, and Thr), and a C-terminal sequence, which is cleaved during the establishment of the GPI (glycosylphosphatidylinositol) anchor in the ER (Schultz et al., 1998). AG-peptides are short classical AGPs with only 10–15 amino acids. Fasciclin-like (FLA) AGPs are also similar to classical AGPs, but possess one or two fasciclin-like domains (FAS) in their protein core (He et al., 2019). Lys-rich AGPs contain a Lys-rich domain between PAST domain and C-terminus, ENODLs contain plastocyanin-like domains, XYLPs contain non-specific lipid transfer protein domains, and HAEs combine modules characteristic for AGPs and EXTs. For further details of classification see recent reviews (Ma et al., 2017; Silva J. et al., 2020).

Proposed mechanisms of AGP functions vary among groups or may be combined within a single protein. Crosslinking of glycoproteins, such as EXTs and AGPs, by cell wall peroxidases might reinforce the cell wall (Bradley et al., 1992; Kjellbom et al., 1997). AGPs are covalently linked to pectins or hemicelluloses (Immerzeel et al., 2006; Tan et al., 2013) and their action as “pectin plasticizers” was hypothesized (Lamport et al., 2006; Corral-Martinez et al., 2019). Another putative mechanism is an enzymatic release of mobile oligosaccharides from AGP glycan side chains that may act as signaling molecules possibly recognized by plasma membrane receptors (Showalter, 2001; Van Hengel and Roberts, 2002; Zagorchev et al., 2014; Silva J. et al., 2020). In spite of studies linking activity of plant chitinases with AGPs action in developmental processes (van Hengel et al., 2001; Dos Santos et al., 2006; Zielinski et al., 2021), this mechanism needs to be proven and membrane receptors recognizing AGP-borne oligosaccharide fragments are not yet characterized. AGPs crosslinked with other cell-wall polysaccharides, especially pectins, can also modulate the plasma membrane-cell wall continuum and cell to cell adhesion (Schultz et al., 1998; Showalter, 2001). FLAs can be involved in crosslinking and cell wall adhesion through the interactions of their FLA domains in the protein core – a mechanism proposed based on their similarity with animal fasciclins and their homophilic interactions, which influence developmental processes (Snow et al., 1989; Elkins et al., 1990). The crosslinking with pectins through PAC (Proline-rich Arabinogalactan protein and Conserved Cysteines) domain is another putative mechanism. This type of interaction was documented for AtAGP31 (Hijazi et al., 2014). The protein even interacted with itself through PAC domain *in vitro* (Hijazi et al., 2014).

Arabinogalactan proteins are often attached to the outer side of the plasma membrane by a GPI-anchor. GPI-anchored proteins act as signal transductors that may enable the targeting of partner receptor-like kinases or modulate ligand recognition specificity as co-receptors (Yeats et al., 2018; Zhou, 2019). A proposed function of AGPs may be related to the cleavage of GPI-anchors, which may generate intracellular messengers or extracellular signals to neighboring cells (Schultz et al., 1998; Showalter, 2001). However, this remains to be conclusively proven. The cleavage of the anchor may also release the plasma membrane from the cell wall matrix, influencing membrane dynamics, including the trafficking of membrane receptors between the plasmalemma and inner compartments (Seifert, 2020). AGPs might act as a cargo linkage/receptor during the endocytosis of extracellular material (Wang et al., 2019). The function of AGPs is likely related to the general adhesive properties of their peripheral carbohydrate moieties, which are Ca^2+^ and pH-dependent (Tan et al., 2018). AGPs are putative calcium capacitors (Lamport and Varnai, 2013; Lopez-Hernandez et al., 2020), which bind Ca^2+^ in a reversible and pH-dependent manner and thus enable Ca^2+^ oscillations and signal transduction (Lamport and Varnai, 2013; Lamport et al., 2018). *Arabidopsis thaliana* mutants with compromised glucuronidation of arabinogalactans and AGPs have reduced Ca^2+^-binding capacity, disrupted calcium wave propagation in roots, and show serious growth defects (Lopez-Hernandez et al., 2020). The complexity of putative functions and available study tools still did not provide consistent insight into physiological aspects of this protein family.

## AGPs in Plant Roots

Arabinogalactan proteins are abundant throughout the plant body, including the roots. Su and Higashiyama (2018) summarized expression data for 130 of 151 *AtAGP* genes (including all subgroups; classical AGPs, AG peptides, FLA, XYPL, PAG, etc.) and many of them were expressed in roots. In *Populus trichocarpa*, 18 of 35 identified *PtrFLA* genes were analyzed by qRT-PCR and all of them were expressed in roots (Zang et al., 2015). In *Triticum aestivum*, all 34 identified *TaFLA* genes were expressed mostly in seeds and roots (Faik et al., 2006). In *Oryza sativa*, 10 of the 69 identified *OsAGPs* were abundantly expressed in roots (Ma and Zhao, 2010). AGP epitopes, localized via an immuno-histochemical approach, appeared differentially in various root tissues: pericycle sectors according to vascular tissue context (Knox et al., 1989; Casero et al., 1998), developing vascular tissues, trichoblasts, atrichoblasts, growing root hairs, root caps, and border cells, for review see Showalter (2001) and Nguema-Ona et al. (2012).

Early experiments with β-Glc-Yariv reagent, which interacts with AGPs, precipitates them from solution and disrupts their activity (Yariv et al., 1967; McCartney et al., 2003), indicated a significant role for AGPs in root growth. The β-Glc-Y-enriched medium strongly reduced growth of both the root and the shoot, but the compound itself accumulated only in root. Shoot growth inhibition thus seems to be a secondary effect of the affected root system (Willats and Knox, 1996). The impaired cell elongation of the cortical cells and the bulging of the rhizodermal cells within the elongation and differentiation zones are the primary effects of the treatment (Willats and Knox, 1996; Ding and Zhu, 1997). The ability of β-Glc-Yariv to trigger cell bulging and disorganization of cortical microtubules in roots of *A. thaliana* was later confirmed by Nguema-Ona et al. (2007). Although not specific for a particular AGP, β-Glc-Yariv highlighted the importance of AGPs in root growth and cell differentiation.

A more focused classification of functional mechanisms comes from the study of particular mutants. Disturbances of polysaccharide metabolism and AGP carbohydrate moieties were associated with reduced primary root growth in *reb1/rhd1* (*root epidermal bulger 1/root hair defective 1*), a galactose biosynthesis mutant of *A. thaliana* (see below) (Baskin et al., 1992; Nguema-Ona et al., 2006). Its phenotype can be suppressed by supplementing growth media with 10 mM galactose, which recovered root cell expansion and anisotropic growth of control (Nguema-Ona et al., 2006). Other evidence supporting the role of AGPs and their sugar moieties in root elongation came from the *mur1* (*murus 1*) mutant of *A. thaliana* with reduced fucosylation (see below), which induces a significant reduction of root elongation, and more interestingly, earlier and more frequent lateral root development (Van Hengel and Roberts, 2002). Developing primordia of *mur1* do not label for fucose-containing epitopes (Freshour et al., 2003). Unfortunately, neither of those experiment identified affected phase of lateral root development.

The protein SOS5/AtFLA4 (SALT OVERLY SENSITIVE 5/FLA ARABINOGALACTAN PROTEIN 4) is one of the best characterized AGP members. *A. thaliana sos5/fla4* mutant, with point mutation in the FAS domain of AtFLA4, displays reduced root growth under high salinity. This phenotype is caused by defected cell expansion (for more details see below) (Shi et al., 2003) and can be suppressed by external ABA application (Seifert et al., 2014). Another non-classical AGP influencing root growth and development is AtAGP30, which is not anchored by GPI into plasma membrane. The *atagp30* mutant of *A. thaliana* fails to initiate adventitious roots from a callus culture, but growth of already established roots, lateral roots and root hairs are apparently unaffected (Van Hengel and Roberts, 2003; Van Hengel et al., 2004). *AtAGP30* transcription starts in the primary root with germination, occurs mostly in the root tip and decreases as tissue differentiate (Van Hengel and Roberts, 2003; Van Hengel et al., 2004). Interestingly, its ectopic overexpression is detrimental for shoot development and stable overexpression transformants are not viable (Van Hengel and Roberts, 2003). A recent study linked AtAGP30 with restriction of cadmium (Cd) entrance and root tip tolerance to this stressor (Jing et al., 2019). It seems that the ability to maintain *AtAGP30* expression under Cd stress is proportional to the level of Cd tolerance (Jing et al., 2019). Unfortunately, it is a pure speculation whether for example Cd retention in the cell wall or membrane protection due to AtAGP30 presence is involved.

AGP presence during lateral root development was indicated by positive antibody labeling in e.g., *Musa* spp. (Wu et al., 2017) or *Solanum lycopersicum* (Sala et al., 2017). However, there are not many reports connecting AGPs with lateral root development. Mutant *atfla1* of *A. thaliana* produces a higher number of lateral roots compared to the wild type, which suggests the role of AtFLA1 in early events of lateral root development (Johnson et al., 2011). The phase of lateral root primordia development (initiation, development and outgrowth) which is affected in *atfla1* and can cause the observed phenotype has not been defined. However, peculiar differences in pericycle division of *atfla1* on callus inducing medium hint at initiation and/or starting divisions. *AtFLA1* expression is not root-specific but was detected in the elongation zone of primary roots, and in the meristem and vasculature of lateral roots (Johnson et al., 2011). Cell division as well as cell expansion were affected also in *atagp19* mutant (Yang et al., 2007; Zhang et al., 2011) resulting in plants with fewer lateral roots, and a smaller vascular cylinder of the primary root due to the lower number of procambial cells. Unfortunately, this is mentioned without any details (Yang et al., 2007), only later commented by Zhang et al. (2011). AtAGP19 along with AtAGP17 and AtAGP18 are members of a subfamily of lysine-rich classical AGPs. AGP19 is abundant in the central cylinder of roots (Yang et al., 2007, 2011). Interestingly, decreasing the arabinogalactosylation of AGPs reduces primary root growth (Gille et al., 2013), but induces longer lateral roots in *A. thaliana* (Ogawa-Ohnishi and Matsubayashi, 2015). It is possible that altered carbohydrate side chains of AGPs modify their ability to crosslink *in muro* resulting in changes to cell wall mechanical properties that manifests during cell expansion and organ growth.

Several other AGPs are linked with root growth. *AtFLA3* is barely expressed in the mature roots of wild-type *A. thaliana*, but its ectopic overexpression stimulates primary root growth and triggers the formation of abnormal root cap cells (Li et al., 2010). In contrast, ectopic overexpression of *AtAGP18* significantly inhibits root growth (Zhang et al., 2011). AtAGP18 regulates megaspore development (Demesa-Arevalo and Vielle-Calzada, 2013) but it is expressed also in roots, mostly in vascular tissues (Yang and Showalter, 2007), and its expression is under the control of ABA (Zhang et al., 2011). The AtAGP18-RNAi silenced lines have a high rate of seed abortion. Root growth phenotype was not observed in the same study but it was not analyzed in details (Acosta-García and Vielle-Calzada, 2004). AtAGP18 would therefore be an interesting candidate for future root-focused studies.

## Root Hairs

Several pieces of evidence implicate some AGPs in the regulation of root hair initiation and growth. Aberrant root-hair development in *atagp21* is connected with contiguous root hair formation and high root hair density (Borassi et al., 2020). AtAGP21 is a part of the brassinosteroid regulatory circuits upstream of GL2 (GLABRA2), RHD6 (ROOT HAIR DEFECTIVE 6) and other downstream transcription factors determining the development of epidermal cells into root hairs. AtAGP21 itself is positively regulated by the BZR1 transcription factor and acts as a suppressor of GL2 (Borassi et al., 2020). A root-hair phenotype similar to *atagp21* also occurs in other *A. thaliana* mutants with altered AGP content, such as O-glycosylation, fucosylation, or arabinosylation of AGPs, e.g., *atagp15*, *hpgt* (Ogawa-Ohnishi and Matsubayashi, 2015; Borassi et al., 2020). The *hpgt1-1 hpgt2-1 hpgt3-1* triple-mutant is defective in O-glycosylation of AGPs due to the disruption of hydroxyproline galactosyltransferase 1–3 and forms longer and more dense root hairs compared to wild-type plants (Ogawa-Ohnishi and Matsubayashi, 2015). O-glycosylation of AtAGP21 is essential for its function, particularly secretion and cellular targeting (Borassi et al., 2020). Contiguous root hair development can also be triggered by β-Glc-Y (α-Man-Y has no effect) crosslinking AGPs and limiting their action in the cell wall, providing additional evidence for the role of AGPs in determining rhizodermal-cell fate in *A. thaliana* (Borassi et al., 2020). Another piece of evidence linking AGPs and root hair growth is a long-hair phenotype of *agp14* mutant of *A. thaliana* (Lin et al., 2011) and a short-hair phenotype of higher-order *glcat14* (β*-glucuronosyl-transferases 14A-C*) mutants of *A. thaliana* with increased AGP contents (Zhang et al., 2020).

The role of AGPs in the determination of rhizodermal-cell fate is further supported by studies on other plant species. In *Zea mays* and *Hordeum vulgare*, specific AGP epitopes were detected on the surface of trichoblasts and root hairs, which differed from those of atrichoblasts (Šamaj et al., 1999; Marzec et al., 2015). Moreover, epitopes detected by LM2, LM14, and MAC207 antibodies, which are normally present at the surface of trichoblasts in *H. vulgare*, were absent in the rhizodermis of barley *root-hairless mutant 1* (Marzec et al., 2015). In *Brassica carinata*, downregulation of *BcFLA1*, encoding a FLA AGP, via CRISPR/Cas9 significantly reduced root-hair length in phosphate-deficient conditions (Kirchner et al., 2018). *BcFLA1* expression was enhanced by Pi deficiency, specifically in the low-P efficient cultivar of *B. carinata*. This cultivar is efficient in Pi uptake and increases the length of root hairs in Pi-deficient conditions considerably (Kirchner et al., 2018).

Interestingly, extensin related modifications of O-glycosylation did affect the root hair growth but not cell fate (Velasquez et al., 2015). Proline-rich extensin-like receptor kinase 13 (PERK13) was shown to provide negative control of root hair growth. *A. thaliana* mutant *rhs10/perk13* (*root hair specific 10/proline-rich extensin-like receptor kinase 13*) has longer root hairs. PERK13 has AGP motifs in its extracellular domain, which may be important for its regulatory function (Hwang et al., 2016). It is proposed that AGP motifs sense the cell-wall integrity, triggering down-stream signal transduction (Cho, 2016). These results taken together indicate that AGPs might affect root hair formation via sensing or modification of cell wall properties, and can participate in signaling pathways controlling root-hair cell fate by an interaction with other proteins or cell wall components, e.g., receptor-like kinases or pectins.

## Root Cell Expansion, Differentiation, and Cell-Wall Properties

As for other plant organs, the growth of roots is determined by cell division, elongation, and differentiation, which are tightly connected with cell wall characteristics. Cell wall composition and mechanical properties are developmentally regulated and respond to environmental factors (Cosgrove, 2005; Caffall and Mohnen, 2009; Somssich et al., 2016; Rui and Dinneny, 2020). Localization of GPI-anchored AGPs on the outer surface of the plasma membrane and their linkage to other cell wall components make them putative linkers of protoplast and the cell wall. β-D-glucosyl units of “active Yariv” reagent (Yariv et al., 1967) bind and precipitate AGPs, disrupting their action. Such treatment, similar to anti-AGP antibodies, induces rearrangement of microtubule cortical arrays in rhizodermal cells within minutes (Nguema-Ona et al., 2007) and stimulates an intense swelling of epidermal cells in the elongation zone in the longer term in *A. thaliana* (Ding and Zhu, 1997; Nguema-Ona et al., 2007). The impaired cell elongation was also observed in cell suspension cultures of *Daucus carota* (Willats and Knox, 1996).

A similar effect of AGPs on cell volume expansion is induced if the AGP glycosylation machinery is affected. Mutations in AGP-specific O-galactosyltransferases lead to defects in cell expansion. The *galt2 galt5* (*hydroxyproline-O-galactosyltransferase 2,5*) mutant of *A. thaliana* has two disrupted AGP-specific galactosyltransferases, which are important for binding the galactose to the protein backbone and initializing O-glycosylation (Basu et al., 2013, 2015). Together with the lower glycosylation status of AGPs, the mutant displays reduced seed-coat cellulose content, swollen root-tip cells, and other root growth defects, e.g., inhibition of root growth, reduction of root hair length and density (Basu et al., 2015). Shorter roots were observed also in the quintuple mutant *galt2 galt3 galt4 galt5 galt6*, but surprisingly this mutant formed longer root hairs compared to wild type (Zhang et al., 2021). All these observations highlight the importance of O-glycosylation in cell growth and cell wall deposition (Basu et al., 2015; Showalter and Basu, 2016). In addition, the disruption of two Golgi-localized exo-β-1,3-galactosidases of glycoside hydroxylase family 43 (GH3) in the *gh43* mutant of *A. thaliana* increases the content of cell-wall bound AGPs and triggers serious defects in root cell expansion and adhesion, e.g., root epidermal cell swelling and loss of anisotropic growth (Nibbering et al., 2020). These exo-β-1,3-galactosidases are putatively involved in the processing of AGPs during their maturation in the Golgi, regulating the length of the β-1,3-galactan backbone of AGPs, and altering the affinity of mature AGPs to other cell wall components (Nibbering et al., 2020).

The connection between AGP glycosylation and regulation of root cell expansion and cell wall properties is highlighted in other studies, where galactosylation and fucosylation are modified, affecting AGPs along with pectins and hemicelluloses. The *A. thaliana* mutant *mur1* with a disrupted GDP-D-mannose-4,6-dehydratase enzyme of the GDP-L-fucose biosynthetic pathway contains less L-fucose in cell walls (Reiter et al., 1993; Bonin et al., 1997). L-fucose is a minor component of AGPs (Silva J. et al., 2020) as well as xyloglucans (Somssich et al., 2016). The L-fucose deficient mutant shows reduced root elongation by more than half compared to the wild type, and swollen root tips. Root growth inhibition is caused by a significant reduction in cell elongation, while the activity of root apical meristem is normal (Bonin et al., 1997; Van Hengel and Roberts, 2002). Alteration of root cell anisotropic growth occurs also in the *reb1/rhd1* mutant (Baskin et al., 1992). Reduced root elongation and bulging trichoblasts observed in this mutant (Baskin et al., 1992; Andème-Onzighi et al., 2002) seem related to altered galactosylation of cell-wall xyloglucans (Nguema-Ona et al., 2006). The mutant has defective UDP D-galactose 4-epimerase enzyme (Seifert et al., 2002) and makes structurally different cell wall xyloglucans, which are devoid of galactose and fucose residues (Nguema-Ona et al., 2006). There is also an obvious link to AGPs and cytoskeletal structures, as the trichoblasts of *reb1/rhd1* have disorganized microtubules and lack AGPs detectable by JIM14 and LM2 antibodies (Andème-Onzighi et al., 2002). However, the functional link is currently not known.

Mutant *dim/dwf1 (diminuto/dwarf1)* of *A. thaliana* in the brassinosteroid biosynthesis gene DIM/DWF1 (Klahre et al., 1998) is strongly affected in cell elongation and has reduced cellulose and lignin content (Hossain et al., 2012). The *dim/dwf1* phenotype correlates with the amount of AGPs in the tissue, highlighting the role of AGPs in cell expansion (Takahashi et al., 1995) and implicating them in an executive part of the brassinosteroid signaling circuit (Jia et al., 2020).

### Stress-Enhanced Developmental Response

Arabinogalactan protein-related growth defects often manifest strongly in the presence of high salinity or other stress factors, and are linked to cell wall integrity, maintenance, and adjustment of mechanical properties (Rui and Dinneny, 2020). Synthesis of L-arabinose, which is incorporated into AGPs, EXTs and some cell wall polysaccharides, depend on the *MUR4/HSR8* (*MURUS4/HIGH SUGAR RESPONSE 8*) Golgi-localized UDP-D-xylose 4-epimerase. Plants of *mur4/hsr8* show a significant reduction of L-arabinose (Reiter et al., 1997; Burget and Reiter, 1999; Burget et al., 2003) and a strong short-root phenotype under salinity, but not in either standard or osmotic stress (mannitol treatment) growth conditions (Zhao et al., 2019). Analysis of *mur4/hsr8* mutant indicates defective cell wall structure but not signaling. This phenotype results in decreased root elongation and also cell-cell adhesion, resulting in epidermal discontinuity and bursting of cells (Zhao et al., 2019). Described defects were rescued by exogenous arabinose, but not glucose or xylose, confirming UDP-Ara biosynthesis consequence and affecting the level of AGP staining in roots (Zhao et al., 2019). Other enzymes affecting cell-wall AGPs are FUT4 and FUT6 (α-1,2-fucosyltransferases 4, 6), which are responsible for their fucosylation. Double mutant of *A. thaliana fut4 fut6* has lower content of fucose and xylose in AGP extracts and short-root phenotype under conditions of salt stress (Tryfona et al., 2014).

The role of AGPs as pectin plasticizers and regulators of cell-wall extensibility under salt stress was proposed rather early (Zhu et al., 1993; Lamport et al., 2006; Olmos et al., 2017). Interestingly, AGPs isolated from roots (and other organs) of the seagrass *Zostera marina* reportedly had specific characteristics, distinguishing them from the AGPs of land plants (high degree of branching, high content of terminal α-L-arabinose), which might enhance the salt tolerance of this marine species (Pfeifer et al., 2020). In *Urochloa decumbens*, AGP epitopes accumulated in root cell walls of after aluminum treatment to maintain cell wall flexibility and increase the high-aluminum tolerance of this tropical grass (Silva T.F. et al., 2020). A recently proposed alternative model of AGP action under salinity stress is their function as carriers, binding Na^+^ ions and transferring them into the vacuole via vesicle trafficking (Olmos et al., 2017).

One of the best characterized AGPs in the context of salinity is SOS5/FLA4. The salt-sensitive mutant *sos5/fla4* of *A. thaliana* has swollen root-tip cells due to abnormal cell expansion occurring under salt stress (Shi et al., 2003). Cell walls of *sos5/fla4* have an altered structure. The pectin-rich middle lamella, essential for intercellular adhesion, is reduced and primary cell walls are thinner and less organized compared to the wild type (Shi et al., 2003). Interestingly, the hypertensive *sos5/fla4* root phenotype under salt stress is milder in ABA-oversensitive mutants and suppressible by exogenous application of ABA (Seifert et al., 2014; Acet and Kadioglu, 2020). The protein might act synergistically with ABA as a putative modulator of ABA signaling upstream of cell wall biosynthesis (Seifert et al., 2014) and independent of the RBOHD and RBOHF (RESPIRATORY BURST OXIDASE HOMOLOG D,F) NADH oxidases (Xue and Seifert, 2015) of the ABA-signaling pathway controlling root growth (Jiao et al., 2013).

Interaction between ABA and SOS5/FLA4 modulates the content of H_2_O_2_ under salt stress (Acet and Kadioglu, 2020), indicating a more significant signaling rather than structural role for SOS5/FLA4. This is consistent with an identical phenotype reported previously for two AGP-specific galactosyltransferases (GALT2 and GALT5), fasciclin-like AGP (SOS5/FLA4) and two leucine rich repeat receptor kinases (FEI1 and FEI2) (Shi et al., 2003; Xu et al., 2008; Basu et al., 2015), which placed those components into a single regulatory pathway (Basu et al., 2016) and derived speculation that SOS5/FLA4 might act as a sensor of conditions in the apoplast via FEI kinases (Turupcu et al., 2018; Seifert, 2021). SOS5/FLA4 tagged with GFP was detected on the plasma membrane, soluble in the apoplast, and in endosomes (Xue et al., 2017). Its C-terminal fasciclin 1 domain (Fas1-2) is essential for its function, possibly involved in molecular interactions. The N-terminal Fas1 domain (Fas1-1) stabilizes proteins in the plasma-membrane (Xue et al., 2017), and it is a putative negative regulator of Fas1-2 binding to FEI1 kinase, which might augment the regulation of root growth according to environmental conditions (Turupcu et al., 2018; Seifert, 2021).

The roles of other individual AGPs and their subtypes still remain to be elucidated, but there is extensive experimental evidence (often coming from organs other than roots) which supports their role in cell wall biochemistry, deposition, and signaling. Modulation of *EgrFLA1,2,3* expression levels in *Eucalyptus grandis* (MacMillan et al., 2010, 2015), *PtFLA6* in *Populus* (Wang et al., 2015), *AtFLA11*, *AtFLA12*, and *AtFLA16* in *A. thaliana* (MacMillan et al., 2010; Liu et al., 2020) altered stem cell-wall polysaccharide composition, cell-wall thickness, and stem mechanical properties. *GhAGP3* and *GhAGP4* are specifically expressed during the transition between cell elongation to the secondary cell wall deposition in developing cotton (*Gossypium hirsutum)* fibers, highlighting their roles during secondary cell wall formation (Liu et al., 2008). In *Physcomitrella patens*, application of AGP binding β-Glc-Yariv or the downregulation of *AGP1* reduced the expansion of the protonema apical cell (Lee et al., 2005).

## Root Interactions With Other Organisms

Roots provide an interface for interaction with rhizosphere biota. AGPs, putative environment-cell-wall-protoplast signal transductors (Seifert and Roberts, 2007), are important components of root exudates and root cell walls, especially in the root-cap and root-associated, cap-derived cells (Vicré et al., 2005; Cannesan et al., 2012; Koroney et al., 2016; Swamy et al., 2016; Driouich et al., 2019) and aid in the formation of the rhizosheath (Galloway et al., 2020). As such, they are likely mediators of root-microorganism interactions, participating in the attraction, recognition, and colonization of roots by beneficial microorganisms as well as in root responses to microbial pathogens (Nguema-Ona et al., 2012, 2013, 2014; Mareri et al., 2018) and parasites (Bozbuga et al., 2018).

### Mutualistic Interactions

AGPs and chimeric arabinogalactan protein-extensins (AGPEs) take part in the mutual interactions between roots and microorganisms. AGP-epitopes were found at arbuscular mycorrhiza symbiotic interfaces (Gollotte et al., 1995; Balestrini and Lanfranco, 2006). The involvement of MtAMA1 (ARBUSCULAR MYCORRHIZA AGP 1) in arbuscular mycorrhiza is indicated by the specific expression of the *MtAMA1* gene exclusively in arbuscule containing cortical cells of *Medicago truncatula* (Schultz and Harrison, 2008). Its mode of operation in the plant-fungi interface is still unknown, but signaling feedback from the cell wall might be anticipated. The authors speculate about a possible coreceptor on the plasma membrane or a mobile signaling molecule after its release from plasma membrane by the cleavage of the GPI anchor (Schultz and Harrison, 2008). Interestingly, two AGP-like (AGL) proteins were identified in the genome of *Glomus intraradices*, with a specific structure not found in plants or non-mycorrhizal fungi. These GiAGLs contain repeat domains that can form polyproline II helices with positively and negatively charged faces. The authors suggest their role in the interaction with host cell wall surface (Schultz and Harrison, 2008). Unfortunately, there are few recent references on this particular topic.

A symbiont as a source of AGPs at the host interface was recorded also from free-living cyanobacteria *Nostoc*, containing a putative AGP peptide genes (classical AGP, AG peptide, and FLA class) and cell surface epitopes responsive to AGP antibodies were detected at the *Nostoc-Gunnera* interface (Jackson et al., 2012). Their discovery suggests that the role of AGPs in the host-symbiont interface might develop from rather ancient cell surface interaction processes and AGP role might evolutionarily originate from very early symbioses (Jackson et al., 2012).

Interaction via AGPs during symbiotic infection by nitrogen-fixing rhizobia has been repeatedly proven, for review see Brewin (2004), Brewin et al. (2008), Nguema-Ona et al. (2013), Rashid (2016). Formation of new lateral root organs - nodules colonized by rhizobia, is a tightly orchestrated process, which is mainly initiated by microbial entry via an infection thread (Coba de la Pena et al., 2017; Ferguson et al., 2019). Rhizobia traveling through infection threads are embedded in a matrix containing AGPEs and other glycoproteins (Rathbun et al., 2002; Brewin, 2004; Reguera et al., 2010). Abnormal infection thread development in *Pisum sativum* mutants (*sym33; sym 40*) is associated with disrupted targeting of AGPEs (MAC265 antibody) exocytosis and authors speculate that this might be correlated with inefficient symbiosome formation in mutants (Tsyganova et al., 2009). Cell wall remodeling that takes place during onset of the symbiosome (Coba de la Pena et al., 2017; Tsyganova et al., 2019) is the potentially affected process. AGPs (localized with JIM1 antibody) are present in the nodule membranes during the maturation of symbiosomes in *Pisum* (Tsyganova et al., 2019). Their significance is still unclear but their presence was not observed in nodules of the *sym31* mutant (Tsyganova et al., 2019) with undifferentiated bacterioids and symbiosome membranes staying in the juvenile state (Borisov et al., 1997). This indicates that AGPs play a role in symbiosome maturation and ontogeny (Tsyganova et al., 2019). AGPs are abundant also in the actinorhizal nodules of *Alnus*, especially during early nodulation stages (Berry et al., 2002).

In addition, *AGP*-encoding genes are upregulated in *Oryza sativa* roots upon colonization by *Piriformospora indica* (Nivedita et al., 2020), a beneficial growth-promoting fungal endophyte that improves salt-stress tolerance in many plant species (Waller et al., 2005; Trivedi et al., 2013). In *Triticum aestivum*, AGP-epitopes (detected by JIM14) occur abundantly in roots infected by *Trichoderma* ssp., a beneficial fungal antagonist of phytopathogens (Basińska-Barczak et al., 2020). These recent observations indicate that AGPs may also promote root interaction with beneficial endophytes.

### Response to Pathogens and Parasites

Analyzing the role of AGPs in root response to pathogens, a suppressive role to early infection by microbial pathogens was demonstrated by AGPs extracted from border cells (BC) of *Pisum sativum* and border-like cells (BLC) of *Brassica napus* (Cannesan et al., 2012). AGPs from BL and BLC attracted zoospores of oomycete *Aphanomyces euteiches* and induced their encystment (loss of the motility due to loss of the flagella). The attraction was far more efficient for *P. sativum* extract in agreement with the fact that *A. euteiches* is the pathogen of *P. sativum* not *B. napus*. Root exudates, but not extracted AGPs, then strongly stimulated their germination (Cannesan et al., 2012). Root-associated, cap-derived cells (BC and BLC) thus act as a blind target, trapping the pathogen (extracellular root trap) and preventing its contact with the root proper (Hawes et al., 2000; Driouich et al., 2019; Ropitaux et al., 2020).

There is also substantial evidence that the composition of AGPs in roots or root exudates changes in response to pathogens or parasites. In *Solanum tuberosum*, AGPs (detected with LM2 and JIM15 antibodies) were upregulated in root exudates in response to elicitors derived from *Pectobacterium atrosepticum*, the pathogen causing soft rot disease in potato (Koroney et al., 2016). In *Musa* spp. roots, AGPs were upregulated by *Fusarium oxysporum* f. sp. *cubense* infection (Wu et al., 2017). Changes in AGP levels occurred in the roots of *A. thaliana* infected by *Plasmodiophora brassiace*, which caused clubroot disease. In this case, AGPs were mostly downregulated, but FLA5 was upregulated together with many cell-wall-modifying enzymes, alpha-expansins in particular (Irani et al., 2018). In the roots of *Glycine max*, repression of *FLA* encoding genes was induced by the fungal pathogen *Macrophomina phaseolina* (Marquez et al., 2018) trying to seize root tissues. Besides microbial pathogens, animal parasites induce changes in root AGP levels as well. In roots of a resistant cultivar of *Glycine max*, the upregulation of *FLAs* is triggered by the attack of root-knot nematodes (Beneventi et al., 2013).

Fluctuation of AGP levels occurs also during the attack of parasitic plant species of *Cuscuta* genus on the host-plant stems. Epidermal contact of *Cuscuta reflexa* stimulates the secretion of AGPs by the host plant, *Lycopersicon esculentum*, to enhance its adhesion to the host stem in the early phase of interaction (Albert et al., 2006). Downregulation of *attAGP* (*attachment AGP)* expression decreased the attachment capability of the parasite (Albert et al., 2006). The presence of AGPs in attachment “cement” was recorded on the surface (holdfast epidermal cells) of *C. campestris and C. japonica* stems (Hozumi et al., 2017) supporting the role of AGPs in parasite-host attachment. Accumulation of AGPs in the tip of developing haustoria appear after penetration of the host stem (Hozumi et al., 2017; Shimizu and Aoki, 2019) and expression analysis of *Cuscuta* developing haustoria identified them as FLAs. On the contrary, the later intrusive growth of *Cuscuta* haustorium triggers the depletion of AGPs in stem tissues facing the attack, which was shown for *Pelargonium zonale* penetrated by *C. reflexa* (Striberny and Krause, 2015). In Orobanchaceae root parasites, AGPs accumulate in the hyaline body, a specialized parenchymatous central core of the parasitic haustorium. The functional significance of this accumulation is, however, unclear (Pielach et al., 2014).

Mechanisms of AGP action in root biotic interaction are still unresolved and puzzling. Several mechanisms were proposed, including the recognition and attachment of microbes, formation of a protective biofilm against degradation of cell wall by pathogenic organisms, or antimicrobial action, for review see Nguema-Ona et al. (2013) and Mareri et al. (2018). In addition, the significance of AGPs in response to pathogens is frequently inconclusive. They may act together with EXTs to modify the cell wall cross-linking in response to pathogens, for review see Rashid (2016). In some studies, EXTs seemed more important. Among others, EXTs rather than AGPs correlated with the resistance to *F. oxysporum* f. sp. *cubense*, in spite of the pathogen-induced changes in AGP levels in *Musa* spp. cultivars (Wu et al., 2017). β-Glc-Y reagent failed to affect the interaction with *Pectobacterium atrosepticum*, although AGPs were upregulated in response to this pathogen in *Solanum tuberosum* roots. Root exudate pre-incubated with β-Glc-Y promoted the growth of the pathogen in a very similar way as non-incubated one (Koroney et al., 2016). Higher levels of AGPs and also EXTs were detected in roots of a *Benincasa hispida* cultivar resistant to *F. oxysporum* f. sp. *Benincaseae* (Xie et al., 2011). Various studies indicate that other cell-wall glycoproteins (EXTs or AGPEs), are at least equally important and change their levels in roots in response to pathogens or symbionts (Shailasree et al., 2004; Plancot et al., 2013; Wu et al., 2017; Castilleux et al., 2020).

Further and more conclusive functional characterization of AGPs roles in root-pathogen interactions thus requires direct evidence based e.g., on modulation of *AGP*-genes expression and analyses of induced phenotypes. There are only few studies revealing the role of individual AGPs in this process. *A. thaliana rat1/agp17 (resistant to Agrobacterium transformation 1)* mutant, defective in arabinogalactan protein AtAGP17, is resistant to *Agrobacterium* transformations of root segments (Nam et al., 1999; Gaspar et al., 2004). In spite of the difficulties with *AtAGP17* transcript detection in roots (Gaspar et al., 2004; Yang et al., 2007, 2011), the protein seems highly abundant in root tissues (Yang et al., 2011). It affects the attachment of *Agrobacterium* to the root surface and modulates the systemic acquired resistance, which allows for successful infection (Gaspar et al., 2004). Two other AGPs, *AtAGP12*, and *AtAGP24*, enhanced their expression in the roots of *A. thaliana* after infection of *Plectosphaerella cucumerina*, a necrotrophic fungal pathogen. AtAGP24-GFP localized in close proximity to plasma membrane and the overexpression of *AtAGP24* strongly increased the susceptibility to *P. cucumerina*, which is evidence for its involvement in the pathogen response (Dobon et al., 2015).

There is also direct evidence of the involvement of a particular *AGP* gene in root defense against animal parasites. The knock-out of *AtAGP8* gene in *A. thaliana* leads to a significantly increased susceptibility toward root-knot nematode *Meloidogyne incognita* (Bozbuga et al., 2018). The susceptibility seems related to the cell wall composition and resistance of root tissue to form specific feeding sites, giant cells. These hypertrophied multinucleate cells re-differentiate from a small number of root cells being pierced by a nematode stylet. Their cells walls contain AGPs and are enriched with highly methyl-esterified homogalacturonans, xyloglucans and arabinans, allowing for plasticity and cell expansion (Bozbuga et al., 2018). Increased susceptibility to root cyst nematode was also observed in *reb1/rhd1* mutant (Baum et al., 2000; Wubben et al., 2004) with lower AGP levels in roots (Ding and Zhu, 1997). Besides the *atagp8* and *reb1/rhd1* mutants, increased susceptibility to nematodes was found in two rhamnogalacturonan I pectin deficient mutants of *A. thaliana* (*arabinan deficient 1,2*), while mutants with suppressed mannan and galactan epitopes (*mannan synthesis-related 1* and β*-galactosidase 5*) were more resistant (Gantulga et al., 2008; Harholt et al., 2012; Wang et al., 2013; Bozbuga et al., 2018).

## Conclusion

Cell-wall localized AGPs work as modulators of cell expansion and differentiation, signal transductors on the cell surface, and effectors of responses to environmental conditions and other organisms. In roots, the multifaceted roles of AGPs are emphasized due to the requirement for high growth plasticity and constant exchange of signals with the environment. The data gained from observing plants with altered expression of *AGPs* or carbohydrate composition of cell wall, immunohistochemical studies, and structural analyses clearly link AGPs and their glycosylation status with cell wall properties, cell expansion and organ growth.

Despite the obvious significance of AGPs, we still have limited information about the roles of individual AGPs in roots and the whole plant. Abundance of AGPs, the complexity of their functions, and their obvious redundancy make this issue challenging. A detailed focus on loss-of-function mutants can move us ahead in understanding the mechanisms of AGP action in roots. Characterization of AGP mutants were summarized in this review alongside other studies on cell wall chemistry to provide an overview of the current state of this topic.

## Author Contributions

DH performed the literature survey, drafted and wrote the manuscript. ET performed the literature survey, drafted and wrote the manuscript, and made the figure and table. AS conceptualized and finalized the manuscript. All authors contributed to the article and approved the submitted version.

## Conflict of Interest

The authors declare that the research was conducted in the absence of any commercial or financial relationships that could be construed as a potential conflict of interest.
